# Association between arginine catabolism and major depressive disorder: A protocol for the systematic review and meta-analysis of metabolic pathway

**DOI:** 10.1097/MD.0000000000021068

**Published:** 2020-07-02

**Authors:** Bing Cao, Runze Deng, Dongfang Wang, Li Li, Zhongyu Ren, Lixin Xu, Xiao Gao

**Affiliations:** a Key Laboratory of Cognition and Personality, Faculty of Psychology, Ministry of Education; b National Demonstration Center for Experimental Psychology Education, Southwest University; c Chongqing University Three Gorges Hospital; d Chongqing Three Gorges Central Hospital; e Chongqing Blood Center; f College of Physical Education, Southwest University, Chongqing, China.

**Keywords:** arginine, depression, metabolism, nitric oxide

## Abstract

**Background:**

Alterations in the levels of arginine and its related catabolic products (ie, ornithine, citrulline, and argininosuccinate) in the urea and nitric oxide cycles were reported to play roles in the pathogenesis of major depressive disorder (MDD). The aim of this meta-analysis study is to explore the associations between arginine with its related catabolic products and MDD, and to discuss the possible role of arginine catabolism in the pathoetiology of MDD.

**Methods:**

This study will be conducted in accordance with the Preferred Reporting Items for Systematic Reviews and Meta-Analyses guidelines. The English language literature published in the databases of PubMed, EMBASE, PsycINFO and Web of Science will be systematically searched. Forest plots will be used to estimate the associations between arginine and its related catabolic products with MDD. Subgroup analysis and meta-regression will also be performed to investigate the source of the potential heterogeneity. Sensitivity analysis will be performed to strengthen the results and to investigate whether any single study would have a significant effect on the results of meta-analysis. Publication bias will be tested for using the funnel plot with Begg test and Egger test. The Newcastle-Ottawa Scale will be applied to assess the risk of bias of observational studies.

**Results:**

An integrated assessment of arginine with its related catabolic products may contribute to predict the risk of MDD.

**Ethics and dissemination:**

The results of associations between arginine with its related catabolic products and MDD will be reported in a peer-reviewed publication. With our findings from this meta-analysis, we hope to provide the most up-to-date evidence for the contributions of arginine and related catabolic products to predict the risk of MDD.

**Systematic review registration:**

The protocol of current meta-analysis has been registered at the Open Science Framework [Available at: https://doi.org/10.17605/osf.io/7fn59].

## 1 Introduction

Major Depressive Disorder (MDD) is 1 of the most common psychiatric disorders and leading causes of global burden of disease, which affected more than 350 million people worldwide.^[1,2]^ The underlying pathoetiology of MDD remains poorly understand with disparate triggers including genetic and/or environmental factors.^[3]^ Accumulating studies have focused on predicting or explaining the onset of MDD and the response of antidepressant treatment through possible biomarkers and their associated metabolic pathway.^[4,5]^ Arginine catabolism regulation has been received increasing attention due to dysfunction of oxidative and nitrosative stress in individuals with MDD.^[6–8]^

**Figure 1:**
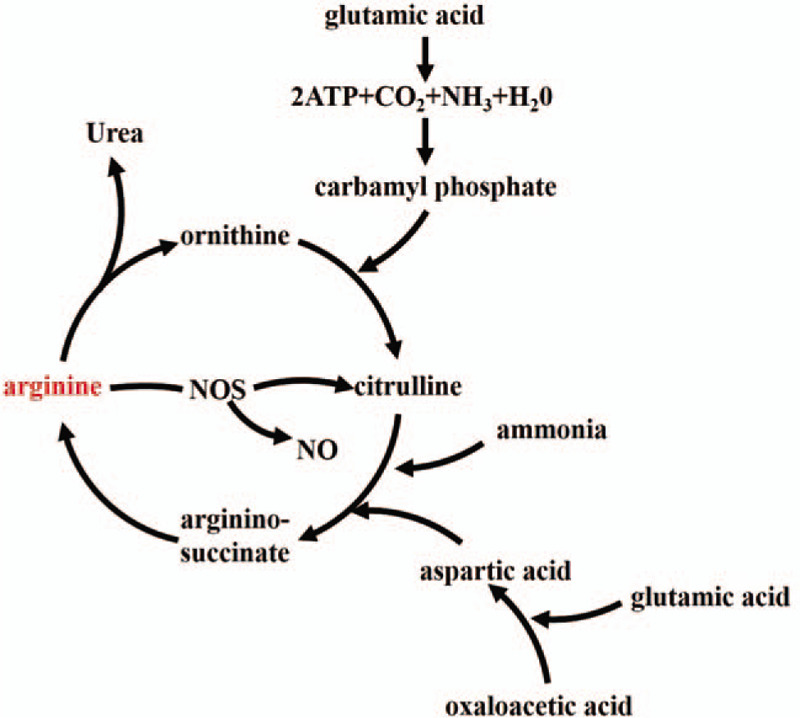
The pathway of arginine catabolism in urea and nitric oxide cycles. Arginine is a substrate for both nitric oxide synthases (NOS), yielding Nitric oxide (NO) and L-citrulline, and for arginase, to produce ornithine and urea. The urea cycle, also known as the ornithine cycle, consists of a series of reactions distributed between the mitochondrial matrix and the cytosol, responsible for the conversion of excess nitrogen into urea. NO is gaseous signaling molecule, which is formed from arginine by NOS. NOS = nitric oxide synthase; NO = nitric oxide; ATP = adenosine triphosphate.

Arginine, a semi-essential amino acid, is reported as a substrate for important pathways to physiological processes in the central nervous system and immune defense such as urea and nitric oxide (NO) cycles^[9,10]^ (Fig. 1). The alterations of arginine might contributed to aberrant NO metabolism and urea cycle pathways.^[11,12]^ Recent cross-sectional studies involving individuals with MDD and experimental study with the rat animal models of depressive-like behavior have reported the associations between dysfunction of blood concentrations of arginine with related catabolic products and NO imbalance and pathophysiology of MDD.^[8,13,14]^ Notably, subjects with MDD are more susceptible to cardiovascular risk factors and are susceptible to comorbidities such as hypertension and diabetes mellitus.^[15,16]^ Moreover, numerous cardiovascular diseases have been reported to associate with NO imbalance.^[17,18]^ Thus, the dysfunction of NO metabolic pathway may establish the link between MDD and it comorbidities through platelet activation, endothelial dysfunction and an elevated concentration of pro-inflammatory circulating cytokines.^[19]^ Arginine was also reported effect levels of dopamine, γ-aminobutyric acid (GABA), and glutamate in prefrontal cortex of brain, which are primarily considered play important roles in the cellular bioenergetics and oxidative stress.^[20]^ Additionally, 2 clinical trials indicated that ketamine and esketamine contributed to the alterations of arginine and other metabolites in the urea cycle.^[21,22]^ Since accumulating evidence has revealed the associations between arginine and its related catabolic products in the urea and NO cycles with MDD, these findings support the role of these metabolites as potential putative diagnostic markers of MDD. However, studies evaluating arginine and its related catabolic products have not clearly illustrated the change pattern between the levels of these metabolites and the MDD.

The aim of this study is to explore the alterations of arginine and its related catabolic products (ie, ornithine, citrulline, and argininosuccinate) in individuals with MDD. The comparisons will be conducted between individuals with MDD and healthy controls (ie, healthy volunteers who were documented to be free from psychiatric problems and histories of mental illness). We hypothesized that an integrated assessment of arginine with its related catabolic products may contribute to predict the risk of MDD.

## 2 Methods

### 2.1 Search strategy

**Figure 2:**
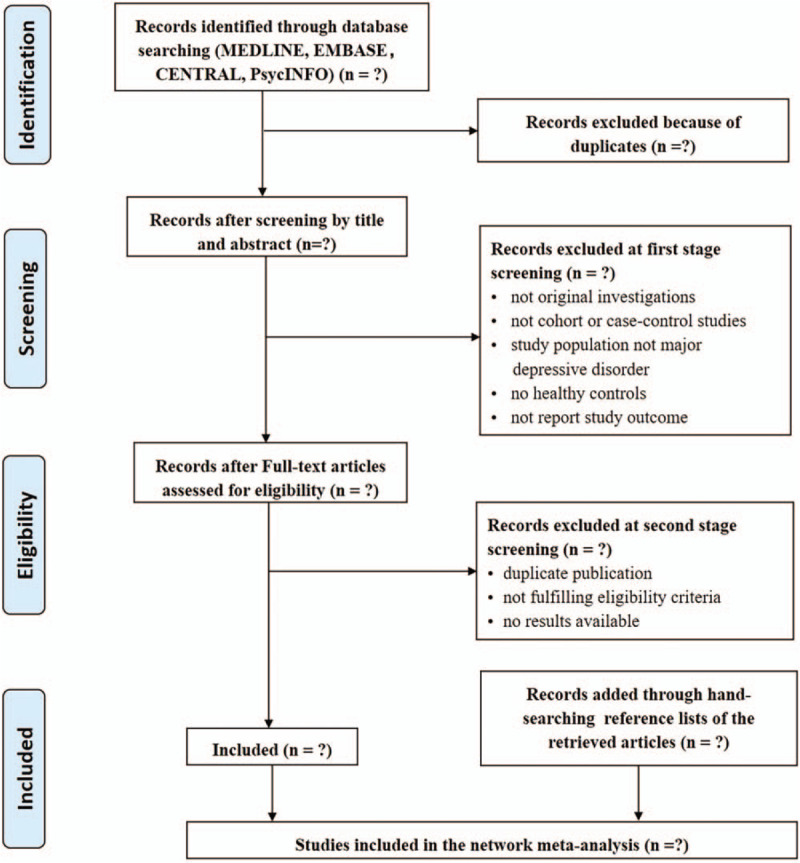
PRISMA flow diagram of study selection process. PRISMA = Preferred Reporting Items for Systematic Reviews and Meta-Analyses.

This systematic review and meta-analysis will be conducted in accordance with the Preferred Reporting Items for Systematic Reviews and Meta-Analyses.^[23]^ Preferred Reporting Items for Systematic Reviews and Meta-Analyses flow diagram of study selection process is shown in Figure 2. We will search the databases of PubMed, EMBASE, PsycINFO and Web of Science for English language literatures. The keywords of our search strategy will be “major depressive disorder”, “depression”, “mood disorder”, “arginine”, “ornithine”, “citrulline”, “argininosuccinate”, and so on. The protocol of current meta-analysis has been registered at the Open Science Framework [Available at: https://doi.org/10.17605/osf.io/7fn59]. The search strategy of PubMed is shown in Table 1.

**Table 1:**
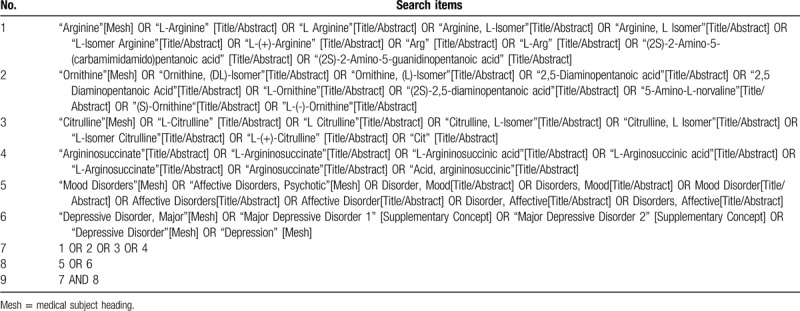
Search strategy for PubMed.

### 2.2 Selection criteria

The case-control or cohort studies meeting the following inclusion criteria will be included in the analysis:

(1) adult subjects (≥18 years old);
(2) assessed a group of unaffected ‘controls’, control subjects were selected from healthy volunteers who were recruited from students, and company employees and so on documented to be free from psychiatric problems and histories of mental illness;
(3) eligible studies included participants meeting the Diagnostic and Statistical Manual of Mental Disorders (no restrictions on editions) or International Classification of Diseases and Related Health Problems criteria for depressive disorders;
(4) the study measured arginine and/or ornithine and/or citrulline and/or argininosuccinate levels among all participants; and
(5) the levels of arginine and/or ornithine and/or citrulline and/or argininosuccinate of participants in the MDD case group and healthy control group were available in the study.

Exclusion criteria of the studies were met if they

(1) did not focus on evaluating the arginine or ornithine or citrulline or argininosuccinate levels on MDD;
(2) only included unhealthy control group with other diseases (eg, bipolar disorder); or
(3) were repetitive publications from the same datasets by the same or different authors.

### 2.3 Outcome measures and data extraction

The outcome for this meta-analysis will be the differences of arginine and its related catabolic products (ie, ornithine, citrulline, and argininosuccinate) between individuals with MDD and healthy controls, as measured by the standard mean differences (SMDs) of their concentrations. Paired investigators will independently select the studies, reviewed the main reports and supplementary materials, extracted the relevant information. All reference lists of the retrieved articles will be reviewed to identify the potential studies. The following information will be extracted from each study: first author, publication year, study design, country, geographic location, age, sex, body mass index, type of blood sample specimen required for test, sample detection method, sample size, subjects’ mean arginine or ornithine or citrulline or argininosuccinate levels, and standard deviations.

### 2.4 Statistical analysis

All the data analyses will be conducted using Stata (version 15.0, Stata Corp LP, College Station, TX). Forest plots will be used to estimate the association between arginine and its related catabolic products with MDD, which will be evaluated by SMD with a 95% confidence interval. The heterogeneity across the studies will be evaluated by chi-square statistics and I-Squared (*I*^*2*^) test, which shows that the percentage of the variability in effect estimates owes to heterogeneity rather than chance. If *P* < .10 or *I*^*2*^>50%, we consider that the heterogeneity had statistical differences and we will use a random effects model. Otherwise, the fixed effect meta-analysis will be applied.^[24]^ According to the statistical power analysis for the behavioral sciences (2nd edition), the effect size (ES) of SMD is judged using the following rules: ’trivial’ (ES < 0.20), 'small’ (0.20≤ ES <0.50), ’medium’ (0.50≤ ES <0.80), and a ’large’ effect (ES≥0.80)”. (Cohen, 1988). Subgroup analysis will be performed to explore the potential influence of included characteristics of the studies on the pooled ES. Meta-regression will also be performed to investigate the source of the heterogeneity, and the effect of both continuous and categorical factors on the study can be assessed simultaneously. The subgroups will be created according to mean or median age, publication date, mean or median body mass index, geographic location, type of blood sample, sample detection method, and so on.

Sensitivity analysis will be performed to strengthen the results and investigate whether any single study would have an effect on the heterogeneity of total measurements in each meta-analysis. The funnel plot with Begg test and Egger test will be used for testing the publication bias. The Cochrane Collaboration recommended the Newcastle-Ottawa Scale (NOS) as an assessment tool for testing the risk of bias for observational studies.^[25]^ The NOS include 3 aspects: participant selection (0 - 4 points), comparability of the study groups (0 - 2 points) and the assessment of the outcome or exposure (0 - 3 points). The total NOS scores categorized into 3 groups: low risk of bias (7 - 9 NOS points), high risk of bias (4 - 6), and very high risk of bias (0 - 3). All 2-tailed *P*-values < .05 will be defined as statistical significance.

### 2.5 Patient and Public Involvement

No patients will be involved in this study.

### 2.6 Ethical approval

All analyses were based on previous published studies, thus no ethical approval and patient consent are required.

## 3 Discussion

### 3.1 Strengths of this study

There are growing interests in the researches of arginine catabolism/NO regulation in the development and progression of MDD. Our current meta-analysis is expected to clarify the associations between 4 metabolites (ie, arginine, ornithine, citrulline, and argininosuccinate), and furtherly illustrate the correlations among these metabolites in the NO regulation pathway of MDD in Figure 1. We hope that the results from our meta-analysis will provide valuable clues to assist researchers who are engaged in the study of basic metabolic mechanisms of MDD.

### 3.2 Potential resources of limitations

Due to the following potential limitations, the results of our meta-analysis may need to be interpreted carefully. First, we might not find a sufficient amount of original researches to perform the analyses. Second, we are focusing on 4 metabolites (ie, arginine, ornithine, citrulline, and argininosuccinate) and perhaps there are not enough reports of each metabolite. Third, our meta-analysis will only include the full-length articles published in English language journals. The data from gray literature (such as unpublished studies and non-peer reviewed literature) or non-English language journals will be excluded. Thus the number of studies might be quite small. Additionally, the potential high heterogeneity caused by differences in age, sex, geographic location, racial, and so on, will lead to selection bias and also decrease the reliability of our results.

To the best of our knowledge, this will be the first meta-analysis exploring the links between the arginine with its related catabolic products and MDD. With our findings from this meta-analysis, we hope to provide the most up-to-date evidence for the contributions of arginine and related catabolic products to predict the risk of MDD.

## Acknowledgments

None.

## Author contributions

**Conceptualization:** Bing Cao, Xiao Gao.

**Data curation:** Bing Cao, Runze Deng, Li Li.

**Investigation:** Li Li, Runze Deng.

**Methodology:** Bing Cao, Dongfang Wang.

**Software:** Bing Cao, Li Li.

**Writing – original draft:** Bing Cao, Runze Deng, Dongfang Wang, Li Li.

**Writing – review & editing:** Xiao Gao, Zhongyu Ren, Lixin Xu.
